# Anticipating Caries

**Published:** 1885-11

**Authors:** C. E. Francis

**Affiliations:** New York


### ANTICIPATING CARIES.

BY C. E. FRANCIS, D. D. S., NEW YORK.

Often has it been laughingly remarked, that if our foresight was equal to our aftersight, the bent of our action would in many instances have been very differently shaped. And how pertinent this quotation, when considering the future welfare of our *clientele*, as regards the preservation of their organs of mastication.

A wonderful faculty, indeed, that would enable one to see clearly into the "dim and shadowy future" and prognosticate with accuracy for years ahead. If this could be done with mathematical precision, many tasks of a perplexing nature would be comparatively easy to manage, or to wholly prevent, and much trouble might be saved. However, lessons gathered from experiences and observations of the past, if carefully weighed and duly considered, may prove of great service in giving character to present decisions.

That teeth in the mouths of some individuals decay more readily than in others is a fact everywhere known, and while many persons have little or no trouble in keeping them in good condition, others must give them unremitting care, or they become a source of perpetual annoyance.

Such varied conditions are due to peculiarities and circumstances. Much depends on shape, arrangement, texture or degree of density, and the physical condition of their possessors. Much also in the way of their preservation depends on proper use, methods of cleansing or degree of cleanliness, a systematic observance of hygienic laws, and by frequent examination, with good care and treatment at the hands of the intelligent dentist.

A crowded condition of the dental arch favors proximal decay, the most vulnerable point being the point of enamel contact, a fact which no practitioner of extended experience has failed to observe. Extraneous accumulations that work their way into the dental interstices are retained in their cervical pockets, and when in a state of fermentation cause mischief to the enamel walls. The poorer the quality of enamel structure, the greater is the danger to be apprehended from agents of decalcification. Teeth of a low-toned or impoverished character, if crowded, are almost always sure to be attacked by proximal decay, and especially is this the case with the superior bicuspids, and with incisors where overlapping occurs.

How to obtain relief where teeth are badly massed together is, in many instances, an exceedingly perplexing undertaking, and a great diversity of opinion exists as to methods of securing it. With young patients, space is often obtained by enlarging the dental arch, by means of regulating appliances. The "Coffin" and "Talbot" devices are valuable aids in accomplishing this result. Room is also frequently secured by extracting one or more teeth, according to requirements. This practice, as a means of anticipating or preventing the formation of caries has, for half a century or more, been earnestly recommended and largely adopted by many prominent members of our calling in this and other countries, and continues to be the practice of a great number of our fraternity who have, with sorrow, witnessed the destruction of many fine dentures which irregular and crowded conditions have tended to hasten. No one will dispute the assertion that the timely removal of a single tooth has, in innumerable instances, been the means of saving several from destruction. Such pioneers of the dental art as Drs. Parmly, Foster, Dunning, and others who practiced in New York, men of excellent professional record, were forced to the conclusion that twenty-eight sound, substantial teeth, requiring little or no care on the part of the dentist, were infinitely better and far more lasting than thirty-two defective ones that demanded constant care, and which were destined to become loaded with metallic fillings or to crumble away—perhaps *both*, in the end. Our esteemed friend, Dr. Clowes, of New York, whose efforts to save human teeth have been crowned with remarkable success, and whose valuable suggestions are based upon a long career of active practice and careful observation, has advocated the same doctrine, which he considers *sound*. Many times, at society gatherings, has he declared that "enamel contact is always a menace of danger," and in order to give relief to a crowded arch he would "weed out" the sickly occupants, and thus secure room and health for the other members of the dental community.

On the other hand, many excellent dentists prefer cutting away the proximal surfaces of the teeth, rather than to obtain space by extraction. With adult teeth of good texture, this practice, in many cases seems to have answered the purpose intended, and has proved the salvation of teeth thus treated. Thousands of dentures have been separated by means of files, disks, and chisels, to save them from decay, and in some instances, although reduced to unsightly slabs, have for years firmly maintained their integrity, as if in utter defiance of the agents of decalcification, or the elements of decay.

Who has not beheld such cases, and plenty of them? Teeth standing alone, unless previously tainted, if treated with any sort of care seldom decay, and mere fragments that have been reduced by artificial means have remained in a state of health where, even in the same mouths, other teeth crowding each other have succumbed to the ravages of disease.

Grains of wisdom may often be gathered by acquainting ourselves with the experiences of others, and in witnessing the results of their practice, and in efforts to prevent proximal decay some points thus gained are worth considering.

As a rule, where the dental arch is unduly contracted, the teeth irregular or crowded, much benefit may be derived by expanding the arch with mechanical devices. This will sometimes accomplish the object desired. If, in irregular and crowded conditions, where the teeth seem impoverished, the enamel rough or abounding in pits, and showing unmistakable evidences of proximal failure, it is good practice to remove either the sixth-year molar or one of the bicuspids on either side of one or both arches, according to circumstances.

Where extraction is the resort for anticipating caries, it should be done sufficiently early in life to obtain the space required. Every year's delay, after the eruption of the second molars, lessens the chances of gain by this means.

One of the principal objections to the removal of the above named teeth is, that a part of the masticating surface is lost thereby. If, however, the loss of this little surface for a brief period of time (or until the spaces close up) will insure a much larger masticating surface for a life-time, is it not simply making a comparatively small sacrifice to gain a permanent benefit? Mastication is no less important to the health and comfort of a mortal as the years of life's march roll on.

If space is to be obtained by cutting away the tooth-structure, it is important to consider the nature of the material to be thus treated, and to do it *artistically.* Compact and yellow teeth in the mouth of an adult will bear the effects of cutting better than the more delicate and pearly-looking ones. Children's teeth are less benefited by having them filed apart, and the expediency of thus treating them is (as a rule) one of serious doubt.

The main objections to cutting away the proximal surfaces are, that their contour is destroyed, and the spaces thus formed are productive of annoyance in masticating food, but it is argued that even this is better than to have them *decay* away.

We confess that we greatly dislike to see teeth mutilated by having their surfaces ground away, and we dislike also to see teeth breaking down from the effects of caries.

But, in all conditions where crowding occurs, *good judgment* is a great requisite when considering what to advise; and good judgment comes only by careful study and close observation. In any event, under all circumstances, a condition of cleanliness within the oral cavity is a most important consideration. Unfortunately, however, so small a proportion of human mortals manage to keep their teeth free from mischievous deposits, that little reliance can be placed on *their* efforts to prevent approximal decay.

---

NOTE.—As this article treats simply of the "anticipation" of caries, the saving of teeth by means of fillings is not herein referred to.

